# Expression, characterization, and immobilization of a novel SGNH esterase Est882 and its potential for pyrethroid degradation

**DOI:** 10.3389/fmicb.2022.1069754

**Published:** 2022-12-21

**Authors:** Wei Zong, Wenfeng Su, Qingfen Xie, Quliang Gu, Xinyi Deng, Yifei Ren, He Li

**Affiliations:** ^1^Key Specialty of Clinical Pharmacy, The First Affiliated Hospital of Guangdong Pharmaceutical University, Guangzhou, China; ^2^Guangdong Key Laboratory of Bioactive Drug Research, College of Life Sciences and Biopharmaceuticals, Guangdong Pharmaceutical University, Guangzhou, China; ^3^Guangzhou Hua shuo Biotechnology Co. Ltd., Guangzhou, China

**Keywords:** pyrethroids, esterase, immobilization, alkali resistance, reusability, bioremediation

## Abstract

The widely-used pyrethroid pesticides have attracted public attention because of their potentials to cause environmental pollution and toxic effects on non-target organisms. Esterase is a kind of hydrolytic enzyme that can catalyze the cleavage or formation of ester bonds. it plays a pivotal role in the decomposition of pyrethroids and esters containing industrial pollutants through the hydrolysis of ester bonds. Here, a new esterase gene est882 was successfully screened, which encodes Est882, a SGNH family esterase composed of 294 amino acids. It was heterogeneously expressed, identified and immobilized. Multiple sequence alignment showed that Est882 had a typical GDS(X) conserved motif and a catalytic triad composed of Ser79, Asp269 and His275. Phylogenetic analysis showed that Est882 shall belong to a new esterase family. Biochemical characterization demonstrated that the optimum condition was 40°C and pH 9.0. Est882 immobilization was studied with mesoporous silica SBA-15 as the carrier and found to significantly improve the tolerance and stability of Est882. Its optimum pH increased to 10.0 and stabilized within pH 8.0–11.0. Free Est882 can effectively degrade various pyrethroids within 30 min, with a degradation rate above 80%. The immobilized Est882 yet degraded more than 70% of pyrethroids within 30 min. The present study indicated that Est882 has outstanding potential in bioremediation of a pyrethroid-polluted environment. These characteristics endow Est882 with potential values in various industrial applications and hydrolysis of pyrethroid residues.

## Introduction

Pyrethroid pesticides are a type of biomimetic synthetic insecticides modified from natural pyrethroids (Fan et al., 2012b). Because of high toxicity to insects and low toxicity to mammals, pyrethroid pesticides are considered to be safer substitutes than the highly toxic pesticides (e.g., organophosphorus) and thus are more widely used (Cycoń and Piotrowska-Seget, 2016). Liu et al. (2020) found that 100 μg/cm^2^ of metofluthrin and transfluthrin could cause significant mortality of mosquitoes within 1 h. According to the research by Kalyan et al. (2010), the percentage of cells with aberration varied from 1 to 32 when lymphocyte cells were treated with varying concentrations of cypermethrin from 3.6 to 7.6 μM (Chakravarthi et al., 2007). Despite agricultural benefits, the extensive and chronic use of pyrethroids has brought many problems, such as drug resistance of pests, toxic effects on non-target organisms (e.g., pest predators, fishes, aquatic invertebrates), abundant residues in agricultural products, water and soils, and human exposure (Yi et al., 2012; Wei et al., 2020). These findings reveal the potential harm of pyrethroids to human health and the ecosystem. Therefore, developing some effective strategies to eliminate or minimize pyrethroid residues in foods and the environment is urgent. Enzyme biotechnology provides safe, efficient and no-secondary-pollution solutions for degradation of residual pyrethroids and other pesticides (Fan et al., 2017). It is critical in bioremediation of contaminated foods, soils, and sewage treatment systems.

The key metabolic routes of pyrethroids are the oxidation by cytochrome P450 and the ester bond hydrolysis by esterases to produce intoxic acids and alcohols (Godin et al., 2007). Esterase is the general name of a class of enzymes that catalyze the hydrolysis or generation of ester bonds, playing a pivotal role in decomposition of pyrethroid compounds and esters containing industrial pollutants through the hydrolysis of ester bonds. Many pyrethroid hydrolases from different sources have been isolated and studied, such as EstPS1, PytY, EstA, Est3385, Est684, and EstP (Wu et al., 2006; Ruan et al., 2013; Cai et al., 2017; Fan et al., 2017; Luo et al., 2018; Hu et al., 2019). However, there are few studies on the alkali resistance and high hydrolysis activity of pyrethroid hydrolases. Most industrial lipolysis and esterification reactions are carried out in the alkaline environment (Kumar et al., 2019; Borges et al., 2020). The ability of hydrolases to survive and catalyze under alkaline conditions is of great importance for industrial applications, especially in bioremediation of pyrethroids-polluted alkaline environments (Wei et al., 2013).

With the development of molecular biology technology, the construction of heterologous expression system has become an important means in the field of industrial enzyme preparation development. Among them, recombinant *Escherichia coli* expression system has the advantages of easy cultivation, fast growth rate and high expression level. It is currently the most commonly used genetic engineering expression system and is widely used in basic research and modern biotechnology. Wang et al. (2009) cloned an esterase gene *pytH* encoding pyrethroid hydrolase from *Sphingobium* sp. JZ-1 and expressed it heterogeneously in *E. coli*. The recombinant PytH has extensive substrate specificity and high hydrolytic activity for various pyrethroids. Zhenqiang et al. (2015) introduced the pesticide-degrading carboxylesterase genes *mpd* and *pytH* into *E. coli* DH5α to construct recombinant plasmids, and integrated them into *Pseudomonas putida* KT2440 for expression. The engineering strain KT2440 could completely degrade six mixed pesticides (0.2 mM each) within 48 h. The construction of engineering microorganisms with broad-spectrum pesticide degradation activity is a promising strategy for bioremediation of pesticide pollution.

Enzyme immobilization technology is an effective strategy to make biotransformation economically feasible (Sheldon et al., 2021). The methods for immobilization are including physical adsorption, covalent, encapsulation or embedding, and crosslinking (Patel et al., 2016, 2017, 2019; Otari et al., 2020). Depending on the immobilization methods, significant variations in the immobilization yields, lower relative activity or loading of enzyme on the supports have been demonstrated. Physical adsorption has simple operation, low cost and maintenance of high enzyme activity, but can very easily desorb enzymes because of the dependence on the weak force between enzymes and the carrier (Cai et al., 2016). Covalent binding usually requires a modifiable and highly stable carrier with more chemical groups, but the operation process is complex (Yang et al., 2021). Although embedding is to fix the enzyme molecules in the grid structure of a porous carrier, and proceeds under mild reaction conditions, the substrate can hardly react with the enzyme due to the influence of the embedding structure. Cross-linking can effectively improve enzyme leakage, but the immobilized enzyme prepared by simple crosslinking has poor mechanical properties, and therefore usually integrated with other methods to strengthen and improve the immobilization effect (Cai et al., 2016; Ke et al., 2018; Yang et al., 2021). Hence, for the success of enzyme immobilization, the selection of appropriate methods is required (He et al., 2015; Cai et al., 2016; Yang et al., 2021). Appropriate immobilization methods are capable of immobilizing free enzymes on a recoverable carrier, so as to reduce production costs and broaden the applications of enzymes (Mogharabi et al., 2012). The application of new carrier such as mesoporous silica nanomaterials endow the immobilized enzyme technology with greater development potential and plasticity, and of them, SBA-15 has large specific surface area, and high thermal and mechanical stability, and thus becomes an ideal candidate for immobilized enzymes (Verma et al., 2020). The quality of immobilized enzymes is improved from all aspects of the immobilization process by using excellent carriers, efficient immobilization methods and process optimization.

In the present study, Est882, a novel alkali-resistant and pyrethroid hydrolytic esterase was discovered from the soils of Junggar Basin in Xinjiang, and its heterologous expression, purification and biochemical characteristics was studied in detail. Then the esterase was immobilized by adsorption crosslinking on SBA-15 as the carrier to improve its stability and reusability. In addition, the biodegradation of fenpropathrin, cypermethrin, permethrin, cypermethrin and fenvalerate by the immobilized enzyme was investigated. The whole research show that immobilized Est882 on SBA-15 may offer a promising technique to increase the degradation of enzyme for pyrethroid pesticides in the future.

## Materials and methods

### Chemicals and reagents

Fenpropathrin, cypermethrin, permethrin, cypermethrin and fenvalerate (purity 98%) were purchased from J&K Scientific Ltd. SBA-15 (pore size 6–11 nm) was bought from XFNANO. T4 DNA ligase, restriction endonuclease and DNA polymerase were provided by Takara. A Bradford protein concentration assay kit was offered by Sigma. All other chemicals were analytically pure commercial products. *E. coli* DH5α and *E. coli* BL21 (DE3) were offered by TSINGKE Biological Technology.

### Prokaryotic expression and purification of recombinant esterase Est882

A soil sample was collected from the wild Ferula asafoetida distribution area in Shihezi on the southern edge of Junggar Basin in Xinjiang. The sample was taken from 5 to 10 cm under the surface, sealed in a sterile bag, and preserved at −20°C until DNA extraction. Total genomic DNA from the soil sample was extracted according to a reported method (Zhao et al., 2021). In the metagenomic library of soil from Junggar Basin, the gene that express the protein with esterase activity was successfully extracted using a genomic DNA extraction kit (Omega, USA). After sequencing, the target sequences were uploaded to National Biotechnology Information Center (NCBI) for registration (GenBank, accession: No.MZ429070) and named as *est882*. The target gene *est882* was amplified by polymerase chain reaction (PCR) with the forward primer TTCCATATGCGAGATCACCGACGTCAGCT and the reverse primer CCC AAGCTTTCCGGCAGGATCTGGAGGTA (the underlined parts are the *Nde*I and *Hind*III restriction sites respectively). The vector pET28a(+) and obtained fragments digested with *Nde*I and *Hind*III were ligated by T4 DNA ligase and the recombinant gene was transformed into *E.coli* BL21 (DE3). The subsequent induction of enzyme protein expression and electrophoresis were conducted referring to previous report (Hårdeman and Sjölin, 2007; Wang et al., 2020). Prior to the phylogenetic tree of esterase was constructed on Mega 7.0 using the adjacency method, the amino acid sequences among Est882 and other esterases were analyzed on Clustal W and ESPript 3.0.

### Enzyme assay

Esterase activity assay was performed with ρ-nitrophenyl (ρNP) esters as substrate. The production of ρNP esters was measured at the absorbance of 405 nm. One unit of esterase activity was defined as 1 μM of ρ-nitrophenol liberated per min under the standard assay conditions (pH 9.0, 40°C, 10 min). The assay was carried out in reaction mixture containing Tris–HCl buffer (50 mM, pH 9.0), substrate solution (1.0 mM) and purified Est882 mixed in a ratio of 18: 1: 1. To eliminate the interference of substrate spontaneous hydrolysis, all experiments were conducted in triplicate (Ksenia et al., 2012; Wang et al., 2020). An enzyme-free mixture was used as the control, and the effect of each substrate on non-enzymatic hydrolysis was subtracted from the measured value of the enzymatic sample (Fan et al., 2012a). In the same group, the highest enzyme activity was set to be 100%, and the relative enzyme activity was calculated.

### Characterization of Est882

The ρNP esters with different acyl chain lengths (ρNP acetate-ρNP myristate, C2-C14) was used as the substrates for specificity test so that the optimum substrate for Est882 can be determined and used for the subsequent experiments. The optimal temperature of Est882 was tested in the range of 10°C–70°C in the solution of pH 9.0. To study the thermal stability, the purified enzyme was cultured in Tris–HCl buffer at 10°C–60°C for certain time (2–10 h), and the residual enzyme activity was detected. The optimum pH of Est882 was determined in the solution of pH 3.0–11.0 at 40°C. The buffer solution (50 mM of final concentration) used for determination of optimal pH contained citrate buffer (pH 3.0–5.0), sodium acetate buffer (pH 5.0–7.0), Tris–HCl buffer (pH 7.0–9.0) and K_2_HPO_4_-KOH buffer (pH 9.0–11.0). The pH stability of the purified enzyme was detected after incubating in different buffers (pH 6.0–11.0) for a certain time (5-25 h). The activities of free Est882 with the addition of optimum substrate to different final concentrations (0.5–5.0 mM) were measured at 40°C in Tris HCl buffer (pH 9.0). The *Km* and *k*_cat_ values were calculated by fitting the data to Michaelis–Menten equation (Wang et al., 2020).

### Immobilization of Est882

Esterase Est882 was immobilized in three different materials including sodium alginate beads (Jampala et al., 2017), chitosan beads with glutaraldehyde as a crosslinking agent (Chunqing et al., 2019) and mesoporous silica SBA-15 (Fan et al., 2017). The process of Est882 fixed on the carrier of mesoporous silica SBA-15 was as follows: SBA-15 (100 mg) was dispersed in 2 ml of citric acid-disodium hydrogen phosphate (CA-DSP) buffer of certain pH, and was added with an appropriate amount of an Est882 solution (1.09 mg/ml of protein concentration, 128 U/ml of enzyme activity). Oscillated at 200 rpm and cultured at room temperature for 1 h, the mixture was added with 0.1 ml of a chitosan solution (dissolved in 5 mg/ml, 0.5% dilute hydrochloric acid-solution) and 0.1 ml of a 1.0% glutaraldehyde solution and was centrifuged to collect the precipitates after further incubation for certain time. To ensure removal of free enzyme, the precipitates were repeatedly washed with CA-DSP buffer 3 times. After vacuum drying at 37°C for 12 h, the chitosan-glutaraldehyde crosslinked SBA-15-immobilized Est882 was obtained (Est882@SBA-15). The enzyme activity recovery of three-carrier-immobilized Est882, namely the activity ratio of immobilized enzyme to free enzyme, was detected for the same time. The immobilization conditions of Est882@SBA-15 were investigated. The optimal pH for immobilization was tested with the CA-DSP buffer at different pH (4.0–9.0). To analyze the effect of immobilization time and enzyme loading, 100 mg of SBA-15 was dispersed in 2 ml of CA-DSP buffer (pH 5.0) with 100–200 μl enzyme solution for certain time (0.5–3 h) at room temperature. 0, 0.1, 0.2 and 0.3 ml of the chitosan-glutaraldehyde solution were each added with 100 mg of SBA-15 for the investigation of the effect on the crosslinking amount. The investigation for substrate specificity of immobilized enzyme was performed in accordance with the free enzyme section above. The optimum substrate was hydrolyzed by reusing the immobilized Est882, and its reuse performance was investigated. Each reaction proceeded for 10 min in the Tris–HCl buffer (40°C, pH 9.0). After centrifugation, washing and drying, the immobilized enzyme was collected, and the fresh reaction mixture was re-added to the next cycle of reaction under the same conditions. Subsequently, the kinetic parameters of immobilized Est882 toward optimum substrate was detected under the same assay conditions as that of free enzyme described above. The residual activity of the immobilized Est882 was tested after 20 consecutive cycles. Referring to previous report (Kumar et al., 2019), the immobilization yield (IY) and efficiency (IE) were calculated as follows:


(1) Immobilization yield%=The total amount of immobilized enzymeThe total amount of enzyme initially added×100

(2) Immobilization efficiency%=Total activity of the immobilized enzymeTotal activity of free enzyme×100

### Material characterization of immobilized Est882

Morphology of the mesoporous silica before and after SBA-15 immobilization was analyzed by a Supra55 scanning electron microscope (SEM, German Zeiss) and a JEM-2100 transmission electron microscope (TEM, JEOL, Japan). SBA-15, free Est882 and Est882@SBA-15 were observed by a NicoletiS10 Fourier transform infrared spectroscope (FTIR, American Thermofisher). An appropriate amount of sample powder was stuck to the conductive adhesive, gold-sprayed for 30 s, and observed under SEM. A proper amount of sample powder was ultrasonically dispersed with anhydrous ethanol and dripped on a copper net. After drying at room temperature, it was observed and photographed under TEM. The sample powder and KBr were mixed evenly at a certain proportion, and made into transparent sheets by pressing the tablet, which were tested on FTIR.

### Gas chromatograph of pyrethroids degraded by Est882

The degradation ability of Est882 over fenpropathrin, cypermethrin, permethrin, cypermethrin and fenvalerate was tested by gas chromatography. The pyrethroids were prepared into a standard pesticide mixture at a final concentration of 5 mg/ml with a pH 9.0 Tris–HCl buffer. The reaction mixture containing 1 ml of standard pesticide mixture and 1 ml of the purified enzyme (1.09 mg/ml) reacted with 3 ml of the Tris–HCl buffer at 37°C for 30 min. The reaction mixture was saturated with sodium chloride and extracted with an equal volume of N-hexane. One microliter of the reaction liquid was used for quantitative analysis by gas chromatograph (GC; Agilent7890B, USA) on HP-5MS column (Chen et al., 2022). The conditions of GC analysis were as follows: the split ratio of 5:1; the injection volume of 1.0 μl; the injection temperature of 260°C; the column flow of 1.0 ml/min. Besides, the carrier gas was high purity nitrogen with a total flow rate of 9 ml/min (Chen et al., 2022). The concentration of pyrethrin was calculated according to the peak area. A0 was used to represent the concentration of pyrethrin of the control group, while those of the experimental group were marked as A1. The calculation formula of the degradation rate (DR) of pyrethrin is shown below:


$$\mathrm{DR}=\frac{\left(A0-A1\right)}{A0}\times 100\%$$

## Results and discussion

### Sequence and phylogenetic analysis

**Figure 1:**
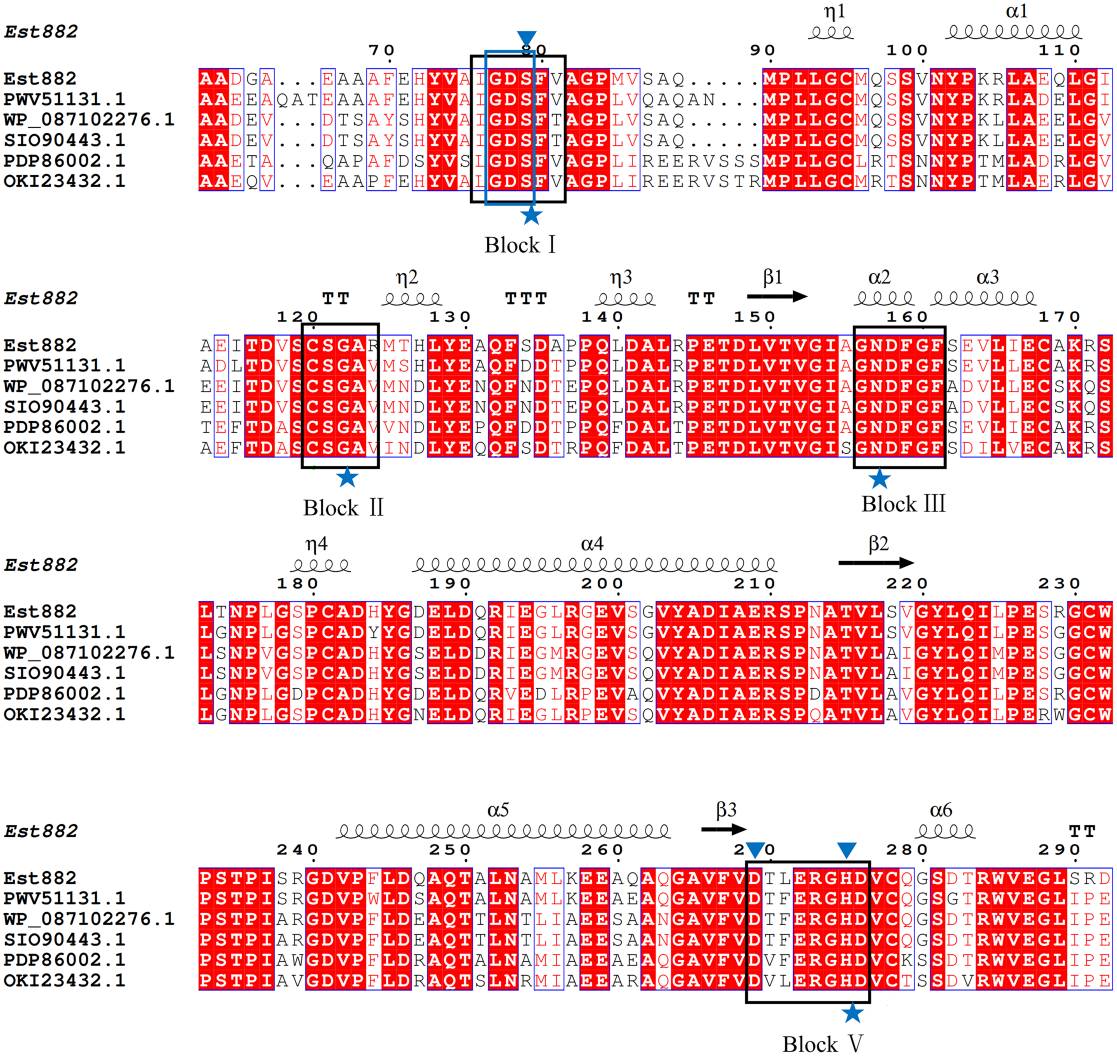
Alignment of multiple amino acid sequences of *Est882* and its homologues. Black rectangles: the four conserved domains (blocks I, II, III and V) of Est882. blue asterisks: the four constant important catalytic residues Ser-Gly-Asn-His in the conserved blocks. Blue rectangle: the conserved GDS(X) motif. Blue triangle: the amino acids (Ser79, Asp269 and His275) that make up the catalytic triad.

**Figure 2:**
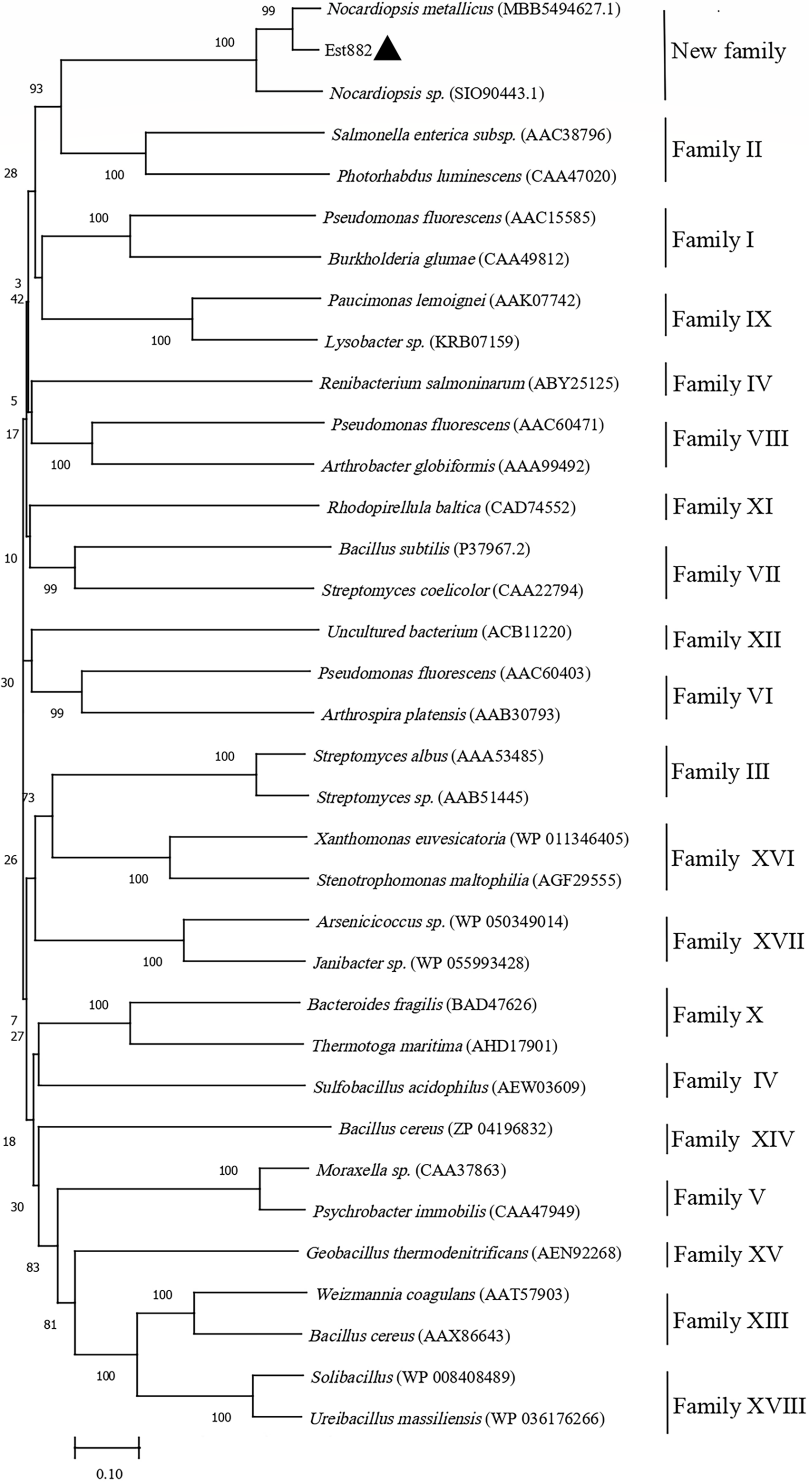
Phylogenetic analysis of *Est882* (▲) and its homologues with other esterases. The amino acid sequences (accession numbers) of all enzymes were retrieved from GenBank.

A new esterase gene was isolated from the soil metagenomic library in Junggar Basin, Xinjiang (Zhao et al., 2021). The esterase gene is in full length of 882 bp and encodes a protein of 294 amino acids (named Est882). The sequence was submitted to GenBank (accession No. MZ429070). The theoretical molecular mass is 31.44 KDa and the isoelectric point is 5.33. Protein blast analysis of NCBI shows that Est882 has some homology with other hydrolases. The encoded protein has the highest homology (88.24%) with the lysophospholipase L1-like esterase from Nocardiopsis metallicus (MBB5494627.1). Multiple sequence alignment with other esterases/lipases shows that Est882 belongs to the SGNH/GDSL hydrolase family, which has a typical conserved motif GDS (X) and a catalytic triad composed of active sites Ser79, Asp269 and His275 (Figure 1). To further validate the evolutionary link between Est882 and other esterases, 32 esterases from 18 families and 2 homologous esterases were chosen to construct an adjacent phylogenetic tree (Figure 2). Phylogenetic analysis did not classify Est882 into the phylogenetic cluster of any known esterase family. This result indicates that Est882 and its homologues may form a new esterase family, so we speculate that Est882 is a new esterase. SGNH family hydrolases have flexible catalytic sites and unique structural properties. Some SGNH family hydrolases have been identified (Song et al., 2013; Petrovskaya et al., 2016; Jo et al., 2021). Most of the hydrolases with GDSL motifs come from plants (Dong et al., 2016; Su et al., 2020). So far, there is no systematic report on SGNH family pyrethroid hydrolases.

### Expression and purification of Est882

**Figure 3:**
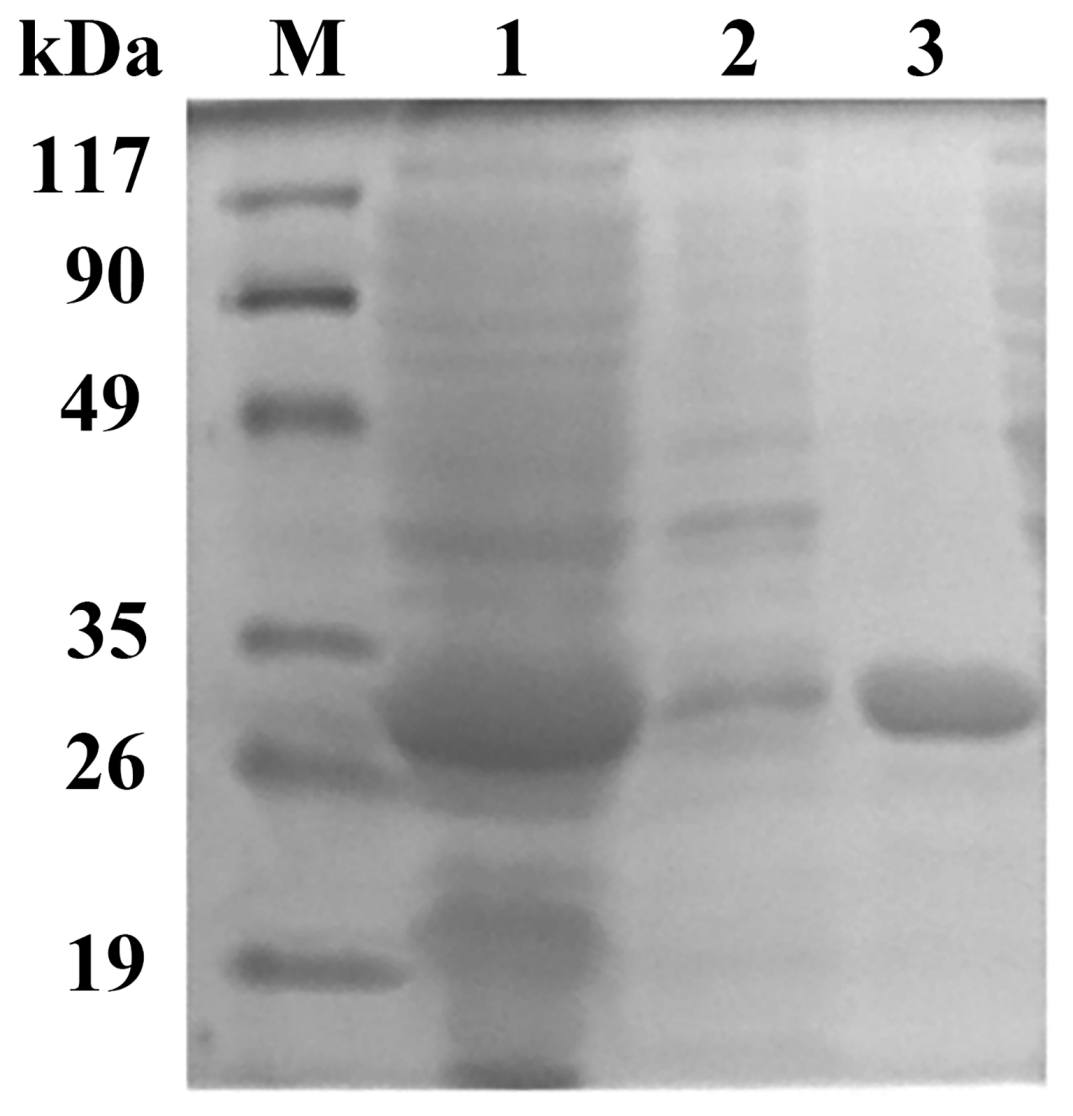
SDS-PAGE of Est882. Lanes M, 1, 2, 3: protein molecular mass marker. Cell lysate. Cell lysate supernatant. and purified *Est882, respectively.*

The target gene *est882* was amplified by PCR (Supplementary Figure S1) and constructed into the vector pET-28a(+). Restriction endonuclease digestion and sequencing confirmed that the target gene was connected to the expression vector (Supplementary Figure S2). The recombinant plasmid pET-28a(+)-*est882* was transformed into *E. coli* BL21 (DE3) for expression. The protein after purification exhibited a single band on sodium dodecyl sulfate polyacrylamide gel electrophoresis (SDS-PAGE; Figure 3). The protein content detected by the Bradford method was 1.09 mg/ml. SDS-PAGE showed the purified enzyme had a relative molecular mass of ~32 kDa, which is consistent with the theoretical molecular mass of the recombinant Est882 (31.44 kDa).

### Substrate specificity

**Figure 4:**
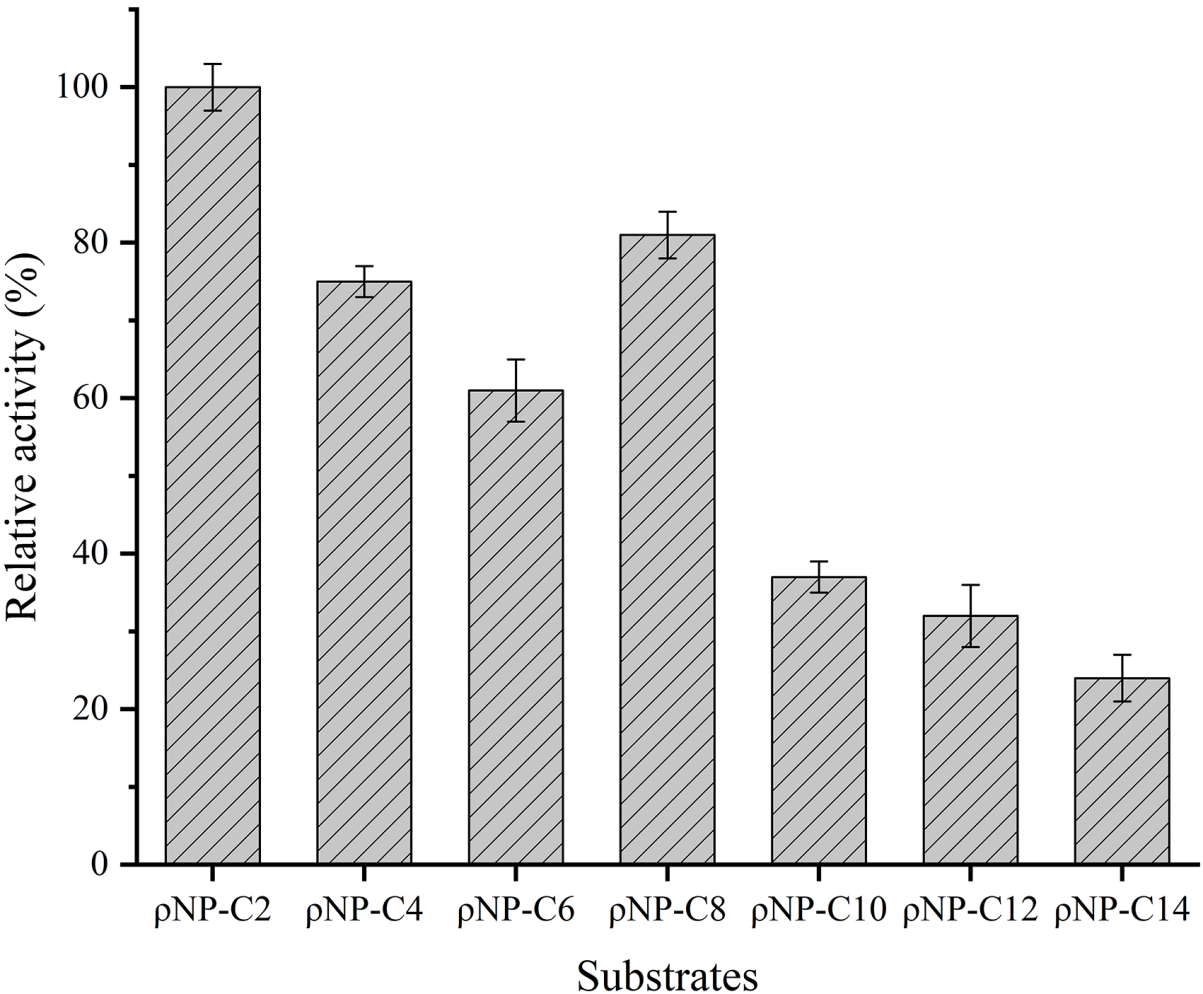
Substrate specificity of Est882. The data point is the average of the three measurements, and the error bar represents the standard deviation.

The substrate specificity of Est882 was determined by using ρ-nitrophenyl esters with different acyl chain lengths as substrates (Figure 4). Est882 displayed the highest activity toward ρNP-C2 among all tested ρ-nitrophenyl esters.

### Effects of temperature and pH on activity and stability of Est882

**Figure 5:**
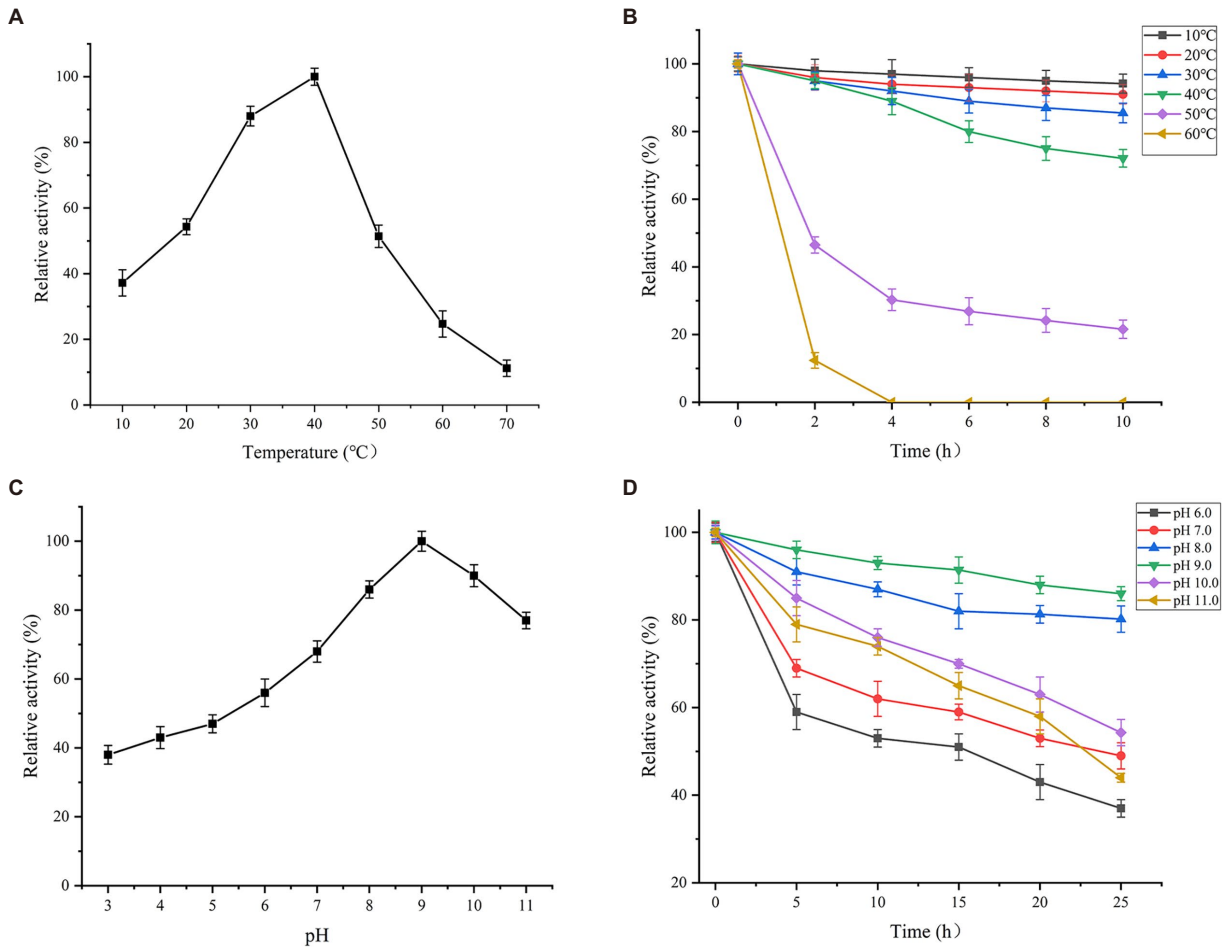
Effects of **(A)** temperature on activity, **(B)** temperature on thermal stability, **(C)** pH on activity, and **(D)** pH on stability of Est882. Symbols: square: 10°C. circle: 20°C. upward triangle: 30°C. downward triangle: 40°C. diamond: 50°C. left triangle: 60°C. Symbols: square: pH 6.0. circle: pH 7.0. upward triangle: pH 8.0. downward triangle: pH 9.0. diamond: pH 10.0. left triangle: pH 11.0.

With ρNP-C2 as the substrate, the effect of temperature on Es882 activity was investigated under standard conditions (Figure 5A). The optimum temperature of Est882 is 40°C. Over 50% of the maximum activity was maintained at 20°C–50°C, indicating Est882 has a wide range of temperature adaptability. Est882 was very stable below 40°C, and the remaining enzyme activity was above 70% after 10 h of incubation (Figure 5B). Similar to Est3385 from *Rhodopseudomonas palustris*. Its optimum temperature is 35°C. When the reaction temperature is 15°C, the relative activity of the enzyme is still about 60% of that at the optimum temperature (Luo et al., 2018).

The effect of pH (3.0–11.0) on the activity of Est882 was tested under standard conditions (Figure 5C). Est882 showed high activity (more than 80%) in pH 8.0–10.0, and the optimum pH was 9.0, indicating Est882 is an alkaline esterase. After incubation in pH 7.0–10.0 for 25 h, the residual activity of Est882 remained more than 50% (Figure 5D), and especially, was more than 80% after 25 h of incubation at pH 8.0 and 9.0. The optimum pH of the enzyme is higher than most reported pyrethroid enzymes, except for the pyrethroid hydrolyzing carboxylesterase EstSt7 from thermophilic S*ulfolobus tokodaii* (pH 9.0; Yi et al., 2012) and the pyrethroid hydrolytic esterases Est684 from Mao-tofu genome and Pye3 from vegetable soil meta-genomic library (pH 7.0; Huang et al., 2016; Fan et al., 2017). The optimum pH of pyrethroid hydrolase Sys410 isolated from Turpan basin metagenomic library is 6.5 (Li et al., 2008; Fan et al., 2012b). Wide temperature stability and strong alkali resistance are important characteristics of hydrolases that can be used to bioremediate changeable environments. Some potential applications of alkaline-resistant enzymes in various industrial productions are reported (Dai et al., 2021). For example, the optimum pH of intracellular azoreductase enzyme from alkaliphilic *Bacillus subtilis* is 7.0 and is still active under alkaline conditions (pH 8.5). It was applied to decompose mixed azo dyes in soils polluted by textile wastewater (Su et al., 2020; Kkp et al., 2021) identified a new β-galactosidase from alkaliphilic *Paracoccus marcusii*, and found its activity remained above 90% after incubation at pH 5.0–9.0 for 3 h, indicating this enzyme is feasible for industrial preparation of prebiotic oligosaccharides. The endoglucanase from *Bacillus subtilis* Y106 has an optimum pH 6.5 and is stable within 4.0–9.0, and thus was applied to pulp modification to improve paper quality (Wang et al., 2017).

### Investigation of immobilization conditions

**Figure 6:**
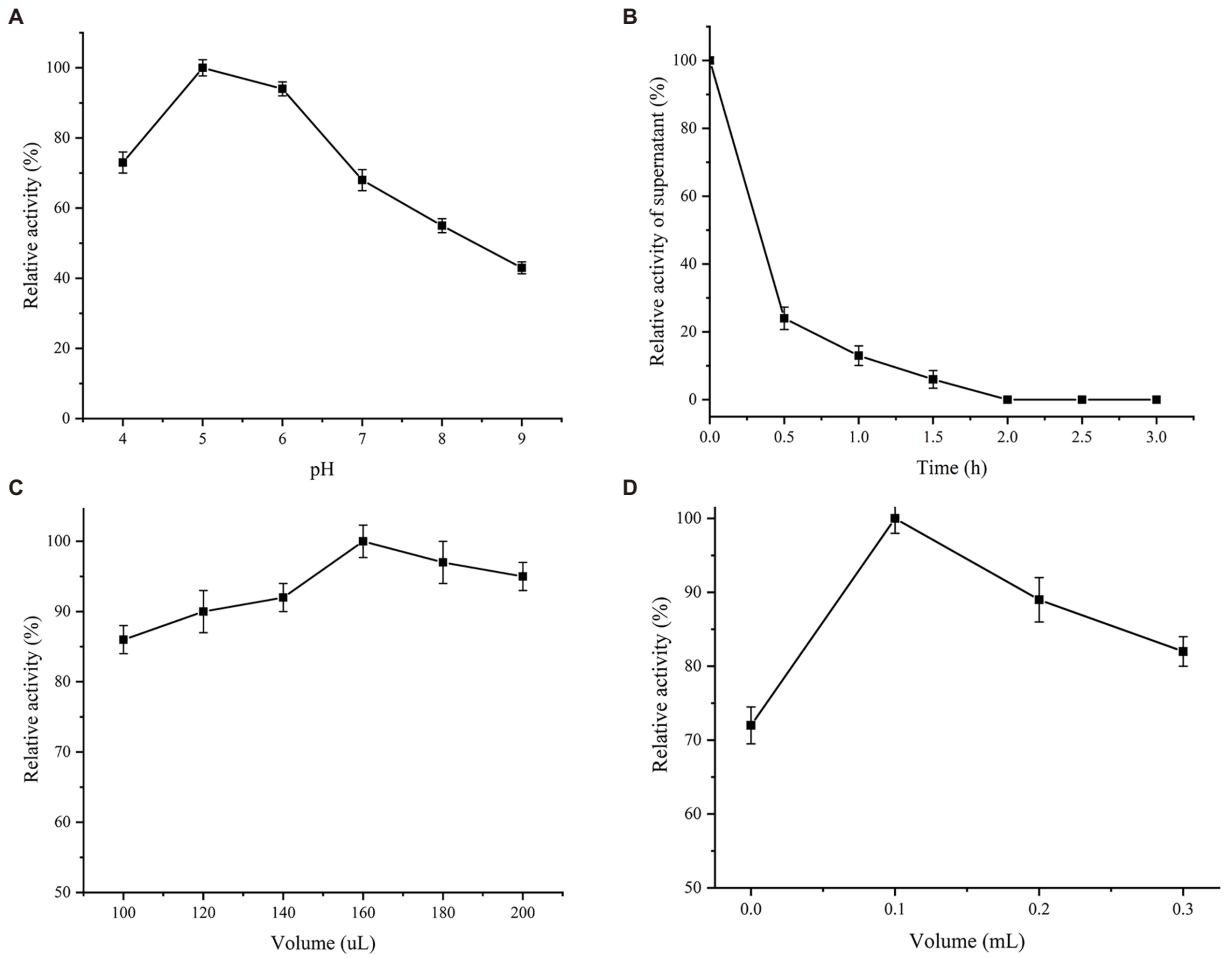
Immobilization conditions of Est882. **(A)** pH. **(B)** time. **(C)** enzyme loading. **(D)** Chitosan addition.

Est882 was immobilized on chitosan, sodium alginate and SBA-15. After the same time, the recovery rates of esterase activity were 57.4%, 42.9%, and 74.2%, respectively. Therefore, SBA-15 was selected as the carrier for further optimization (Table 1). As for the effect of buffer pH, the activity of immobilized enzyme was the highest at pH 5.0, but too high pH was unfavorable for the immobilization (Figure 6A). The immobilization effect of SBA-15 on enzyme was mainly influenced by solution pH, which can be associated with the difference of isoelectric point between enzyme and carrier. Different with the predicted isoelectric point 5.33 of Est882, that of SBA-15 is between 3.0 and 4.0 according to the previous studies that have been shown (Mehta et al., 2016; Zhong et al., 2019). In the solution of pH 5.0, the immobilization of enzyme and carrier presented better combination through the electrostatic interaction of physical adsorption when the carrier and the enzyme have opposite charges. In terms of immobilization time (Figure 6B), Est882 was rapidly adsorbed to the carrier within 1–2 h, the enzyme activity in the supernatant remained low after 1 h, and all the enzyme was immobilized on the carrier after 2 h. The loading amount of 100 mg SBA-15 immobilized enzyme was investigated (Figure 6C). When the enzyme dosage was 160 μl (protein concentration 1.09 mg/ml), the immobilized enzyme activity was the highest, and the optimal loading amount of carrier immobilized enzyme was 1.74 mg/g. Moreover, the optimal crosslinking amount of chitosan glutaraldehyde was 1 ml/g (Figure 6D).

**Table 1:** The activity recovery of the immobilizing *Est882* on different carriers.

Carriers	The activity recovery (%)
Chitosan beads	57.4 ± 1.2
Sodium alginate	42.9 ± 0.7
Mesoporous silica SBA-15	74.2 ± 1.5

### Material characterization of immobilized Est882

**Figure 7:**
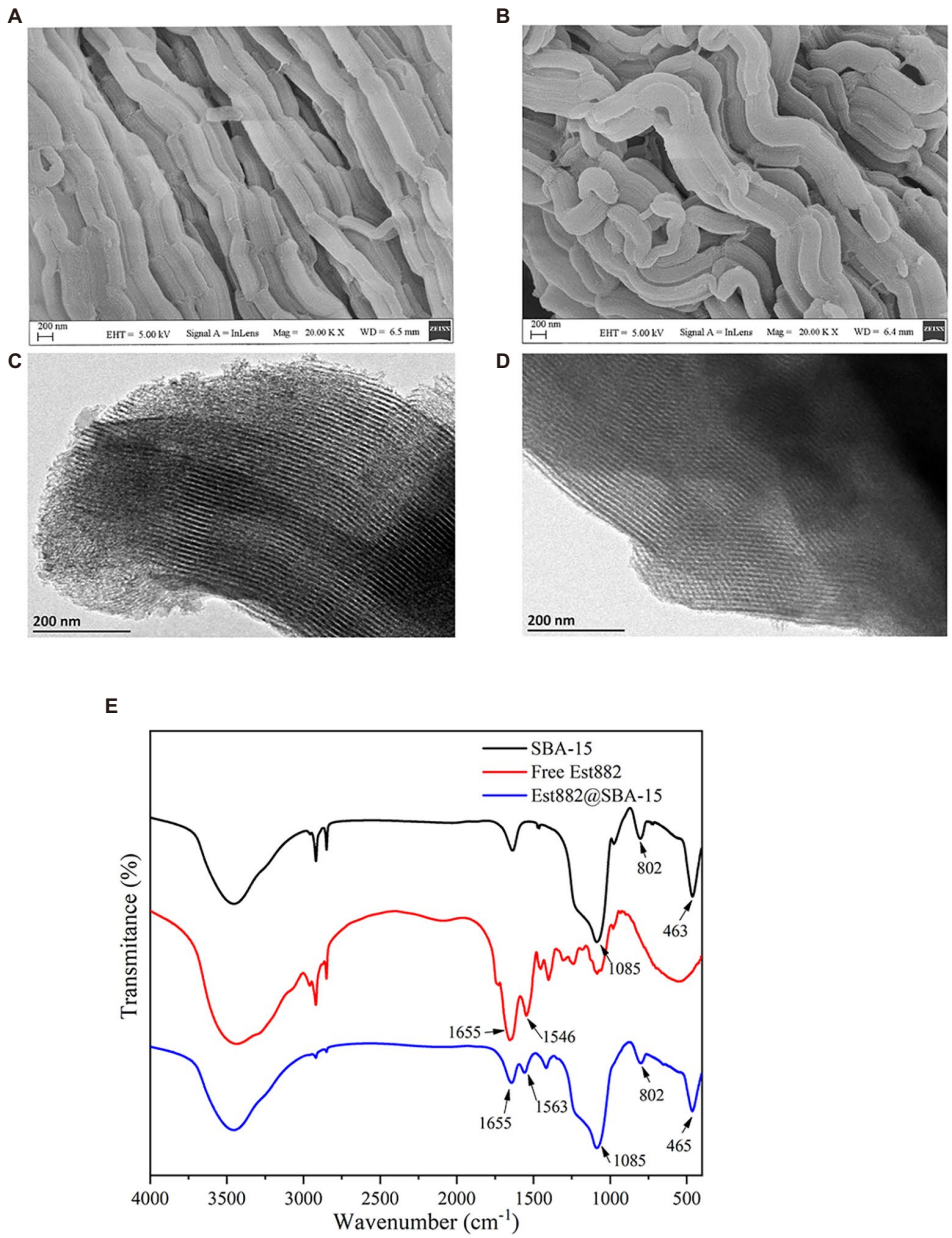
SEM **(A,B)**, TEM **(C,D)**, and FTIR **(E)** of SBA-15 immobilized Est882.

Morphology of the SBA-15-immobilized enzyme was characterized by SEM and TEM. The SEM patterns show worm-like arrays with a certain length (Figures 7A,B). Morphology of the carrier did not change after enzyme immobilization, indicating SBA-15 was still stable after the immobilization. The TEM diagram shows that SBA-15 has uniform and ordered long-channel mesoporous structures (Figures 7C,D), and some opaque shadows appear on the surface after the immobilization, suggesting the enzyme molecules stay in the channel of the mesoporous material, which is also related to chitosan crosslinking.

FTIR demonstrates the characteristic absorption peaks at 463, 802 and 1,085 cm^−1^ of SiO_2_ in SBA-15 (Figure 7E), which belong to bending, symmetric stretching and anti-stretching vibrations of Si-O-Si (Zhong et al., 2019). The typical peaks of free Est882 at 1655 and 1,545 cm^−1^ are ascribed to amide I band (C=O stretching vibration) and amide II band (N-H stretching and C-N bending vibrations). The spectrum of SBA-15 shows obvious characteristic bands of amide I band and amide II band, and bending and stretching vibrations of Si-O-Si, indicating the successful loading of Est882 on SBA-15 (Carlsson et al., 2014).

### Kinetic parameters

The kinetic parameters of the free and the immobilized Est882 were tested using ρNP-C2. The *Km* and *k*_cat_/*Km* are 0.76 mM and 468.17 s^−1^·mM^−1^ in the free enzyme, and 0.85 mM and 272.70 s^−1^·mM^−1^ in the immobilized enzyme, respectively, implying the *Km* of the immobilized Est882 is slightly larger. Compared with pyrethroid-degrading esterase PytY from *Ochrobactrum anthropi* whose *Km* was 2.34 mM (Zhiyong et al., 2013) and pyrethroid-hydrolyzing carboxylesterase EstSt7 from *Sulfolobus tokodaii* whose *kcat/Km* was 246.3 s^−1^·mM^−1^ (Wei et al., 2013), Est882 exhibited a greater affinity for ρNP-C2.

### Comparison of catalytic properties

**Figure 8:**
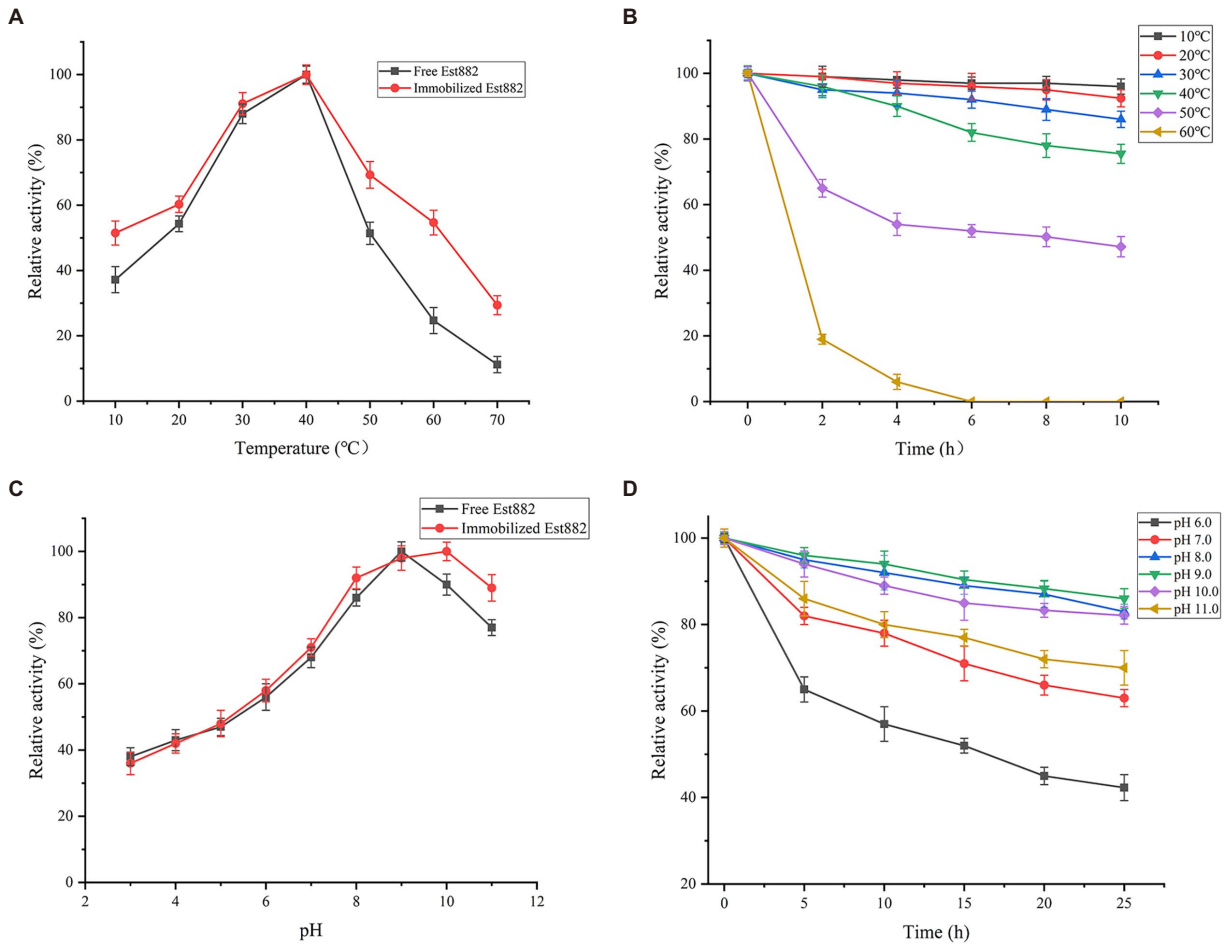
Effects of **(A)** temperature and **(C)** pH on the activity of free Est882 (square) and immobilized Est882 (circle). **(B)** Thermal stability and **(D)** pH stability of immobilized Est882. Symbols: square: 10°C. circle: 20°C. upward triangle: 30°C. downward triangle: 40°C. diamond: 50°C. left triangle: 60°C. Symbols: square: pH 6.0. circle: pH 7.0. upward triangle: pH 8.0. downward triangle: pH 9.0. diamond: pH 10.0. left triangle: pH 11.0.

The activity of the immobilized Est882 was determined in 10–60°C (Figure 8A). The optimum temperature of the enzyme with or without immobilization was 40°C, but the relative enzyme activity at 10–60°C was more than 50% in the immobilized Est882, but that at 60°C was only 24.7% in free Est882. When at above 40°C, the thermal stability of the immobilized enzyme was significantly higher than that of the free enzyme (Figure 8B). After incubation at 50°C for 10 h, the residual activities of the immobilized and free enzymes were 47.2 and 21.6%, respectively.

The optimal pH of immobilized Est882 increased to pH 10.0 from that of free Est882 (pH 9.0; Figure 8C). The stability of pH was investigated after incubation in different buffers (Figure 8D). The residual enzyme activity of immobilized Est882 remained above 60% after incubation at pH 7.0–11.0 for 25 h, and especially was 82.1% in the pH 10.0 buffer compared with the free enzyme (54.3%).

Enzyme immobilization was applied to pyrethroid hydrolase to develop biocatalysts with enhanced stability and reusable efficiency (Cai et al., 2016). In this study, esterase Est882 was immobilized on SBA-15 by adsorption-crosslinking, and the recovery rate of enzyme activity increased to 86.5% under the optimum immobilization conditions. The optimum pH of immobilized Est882 increased from 9.0 to 10.0, and it was more tolerant to alkaline pH. Similarly, Zhang et al. (2012) used SBA-15 to immobilize laccase, and improved the optimal pH from 4.0 to 5.0. Eldin et al. (2000) immobilized penicillin G acylase on methylmethacrylate, and raised its optimum pH from 6.0 to 9.0.

### Reusability of immobilized Est882

**Figure 9:**
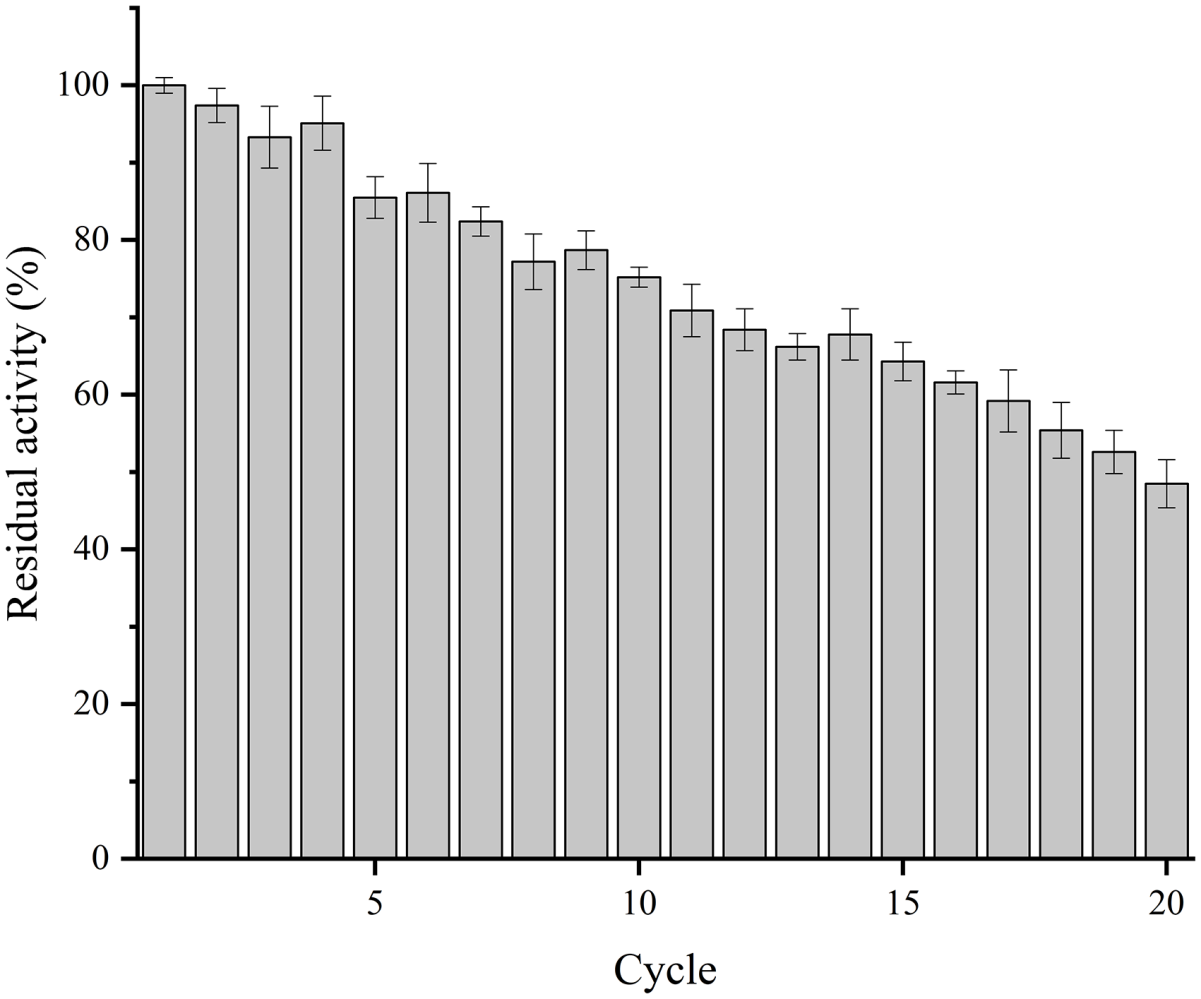
Reusability of immobilized Est882.

The reuse performance of immobilized Est882 was studied for 20 cycles (Figure 9). It was highly stable in the first 10 cycles and retained more than 75% of the initial activity. After 15 and 20 cycles, 64.3 and 48.5% of the initial activity were maintained, respectively. Its reusability is higher compared with the feruloyl esterase immobilized on mesoporous silica by physical adsorption after 10 cycles (44%; Eldin et al., 2000) and the alkaline hydrolase PA27 immobilized in ammonium sulfate by glutaraldehyde cross-linking after 10 cycles (70.2%; Jang et al., 2014). However, it is not as stable as acetylesterase LaAcE, which was crosslinked with glutaraldehyde and immobilized by ammonium sulfate and retained about 85% of its original activity after 10 cycles (Wang et al., 2018).

### Gas chromatograph of pyrethroids degraded by Est882

**Figure 10:**
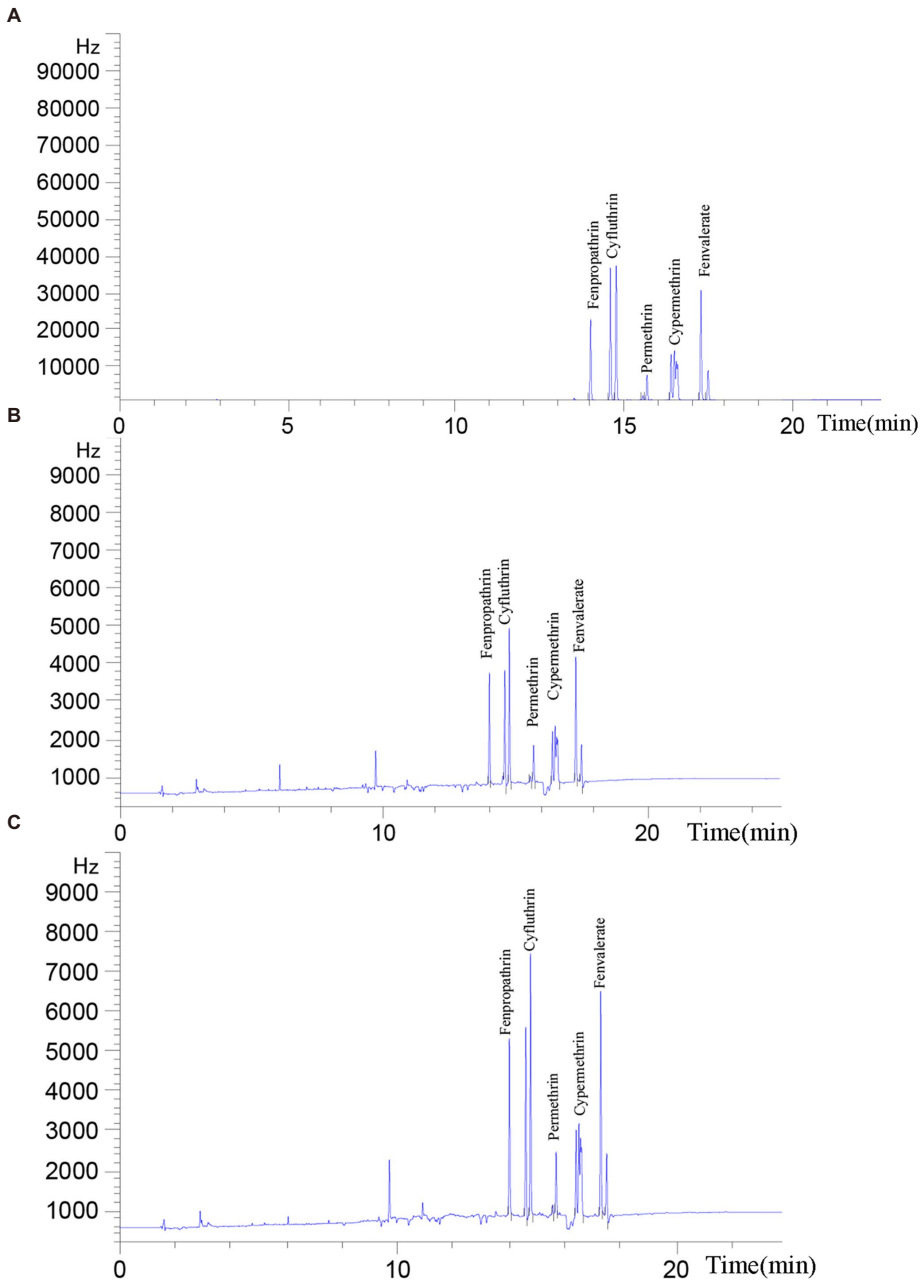
Degradation of pyrethroids by purified Est882. The reaction proceeded at 37°C for 30 min, and a pyrethroid solution containing inactivated Est882 was used as a control **(A)** Ultimate pyrethroid concentrations analyzed by GC after incubation with **(B)** free Est882 and **(C)** immobilized Est882 for 30 min at 37°C.

Five common pyrethroids were used as substrates to investigate the degradation ability of Est882. Gas chromatograph showed that Est882 can effectively hydrolyze various pyrethroids. After 30 min of reaction at pH 9.0 and 37°C, the control without enzyme was incubated under the same conditions (Figure 10A). The hydrolysis rates of free Est882 over fenpropathrin, cyhalothrin, cypermethrin, cypermethrin and fenvalerate were 86.2%, 90.3%, 84.9%, 87.6%, and 88.3%, respectively (Figure 10B; Table 2). The hydrolysis rates of the five pyrethroids by immobilized Est882 were 78.4%, 83.0%, 76.2%, 81.6%, and 79.1%, respectively (Figure 10C; Table 3). Compared with free enzyme, the degradation rates of pyrethroid pesticides by the immobilized enzyme decreased slightly, but still maintained about 89% of the degradation efficiency of the free enzyme.

**Table 2:** Degradation rate of five pyrethroids by Est882.

Pyrethroids	Initial concentration (mg/L)	The concentration after degradation (mg/L)						Degradation rate (%)
		1	2	3	4	5	Mean	
Fenpropathrin	0.991	0.132	0.129	0.145	0.134	0.140	0.136	86.2%
Cyhalothrin	0.984	0.089	0.092	0.105	0.091	0.096	0.095	90.3%
Cypermethrin	0.989	0.154	0.158	0.142	0.151	0.142	0.149	84.9%
Cypermethrin	0.987	0.125	0.130	0.119	0.124	0.112	0.122	87.6%
Fenvalerate	0.983	0.119	0.117	0.102	0.113	0.124	0.115	88.3%

**Table 3:** Degradation rate of five pyrethroids by Est882@SBA-15.

Pyrethroids	Initial concentration (mg/L)	The concentration after degradation (mg/L)						Degradation rate (%)
		1	2	3	4	5	Mean	
Fenpropathrin	0.991	0.221	0.192	0.204	0.231	0.223	0.214	78.4%
Cyhalothrin	0.984	0.162	0.159	0.180	0.165	0.173	0.168	83.0%
Cypermethrin	0.989	0.227	0.235	0.238	0.251	0.225	0.235	76.2%
Cypermethrin	0.987	0.192	0.175	0.184	0.172	0.188	0.182	81.6%
Fenvalerate	0.983	0.215	0.192	0.209	0.210	0.201	0.205	79.1%

Enzyme bioremediation is considered as a potential method for rapid degradation of pesticides, and a growing number of pesticides degrading enzymes are reported (Le et al., 2019). Hu et al. (2019) investigated the degradation ability of carboxylesterase EstA from *Bacillus cereus* BCC01 to various pyrethroids (20 mg/l), and found the degradation rates after 2 h of reaction were more than 70.4%, especially for cypermethrin (100%). Zhang et al. (2021) treated cypermethrin-contaminated vegetables (500 mg/L) with esterase from *Bacillus licheniformis* B-1, and found the degradation rate of cypermethrin residues exceeded 50% at 25°C after 20 min. The pyrethroid hydrolase Sys410 isolated and identified by Fan et al. (2012b) with a metagenomics method had high degradation ability over cypermethrin, cypermethrin, fenpropathrin and deltamethrin (5 mg/ml), and the degradation rates exceeded 95% at 37°C for 15 min. Our study showed the hydrolysis rates of Est882 over cypermethrin, cyhalothrin, cypermethrin, cypermethrin and fenvalerate were more than 80% after 30 min at 37°C, indicating Est882 is a broad-spectrum pyrethroid hydrolase with high hydrolytic activity. The hydrolysis rate of pyrethroids by immobilized Est882 is more than 70%, which is slightly lower than that of the free enzyme. The difference may be due to the fact that enzyme molecules immobilized in mesoporous materials reduce the enzyme and substrate contact, thus slightly decreasing the hydrolysis efficiency of pyrethroid pesticides. Nevertheless, the higher reusability and stability still endow the immobilized Est882 with broad application prospects in the degradation of pesticide residues in vegetables and fruits, treatment of pesticide-containing wastewater, remediation of contaminated soil and environmental monitoring, and become another direction for the development and utilization of pesticide-degrading enzymes in the future.

## Conclusion

Esterase has been widely used in a series of industrial fields, especially in the bioremediation of pesticide pollution. In this study, the gene est882 was found and studied from the metagenomic library constructed earlier by our research team, The gene encodes an esterase of the SGNH family consisting of 294 amino acids. Est882 has a typical GDS (X) conserved motif and a catalytic triad composed of Ser79, Asp269 and His275. Phylogenetic analysis showed that Est882 shall belong to a new esterase family. Est882 had the highest enzyme activity at 40°C and pH 9.0. The pH stability and operational stability of the immobilized enzyme were significantly improved by immobilizing Est882 on mesoporous silica SBA-15 using the adsorption-crosslinking method, and more than 75% of the initial activity was maintained after 10 cycles. The enzyme can effectively degrade several pyrethroids in a very short time, with a degradation rate of more than 80%, showing broad substrate specificity and catalytic activity. The study of Est882, a novel esterase with broad-spectrum and efficient degradation ability, has enriched the SGNH family esterase gene resources, and the combination of favorable properties of Est882 and immobilization process provides a promising pathway for the biodegradation of pyrethroids in contaminated environments.

## Data availability statement

The original contributions presented in the study are included in the article/Supplementary material, further inquiries can be directed to the corresponding authors.

## Author contributions

WZ: conceptualization, original draft preparation, and editing. WS and QX: manuscript reviewing. QG and XD: supervision. HL and YR: project administration and supervision. All authors contributed to the article and approved the submitted version.

## Funding

This study has been funded by Natural Science Foundation of China (grant number 31400680), Science and Technology Plan Project of Guangzhou (grant number 201802030009). And it was supported by National Key Clinical Specialty Construction Project (Clinical Pharmacy) and High Level Clinical Key Specialty (Clinical Pharmacy) in Guangdong Province.

## Conflict of interest

Author YR was employed by “Guangzhou Hua shuo Biotechnology Co. Ltd.”

The remaining authors declare that the research was conducted in the absence of any commercial or financial relationships that could be construed as a potential conflict of interest.

## Publisher’s note

All claims expressed in this article are solely those of the authors and do not necessarily represent those of their affiliated organizations, or those of the publisher, the editors and the reviewers. Any product that may be evaluated in this article, or claim that may be made by its manufacturer, is not guaranteed or endorsed by the publisher.
