# Supplementary figures and images for: Expression, characterization, and immobilization of a novel SGNH esterase Est882 and its potential for pyrethroid degradation
Source: Front Microbiol. 2022 Dec 21;13:1069754. doi: 10.3389/fmicb.2022.1069754 (PMC9810817; doi:10.3389/fmicb.2022.1069754)

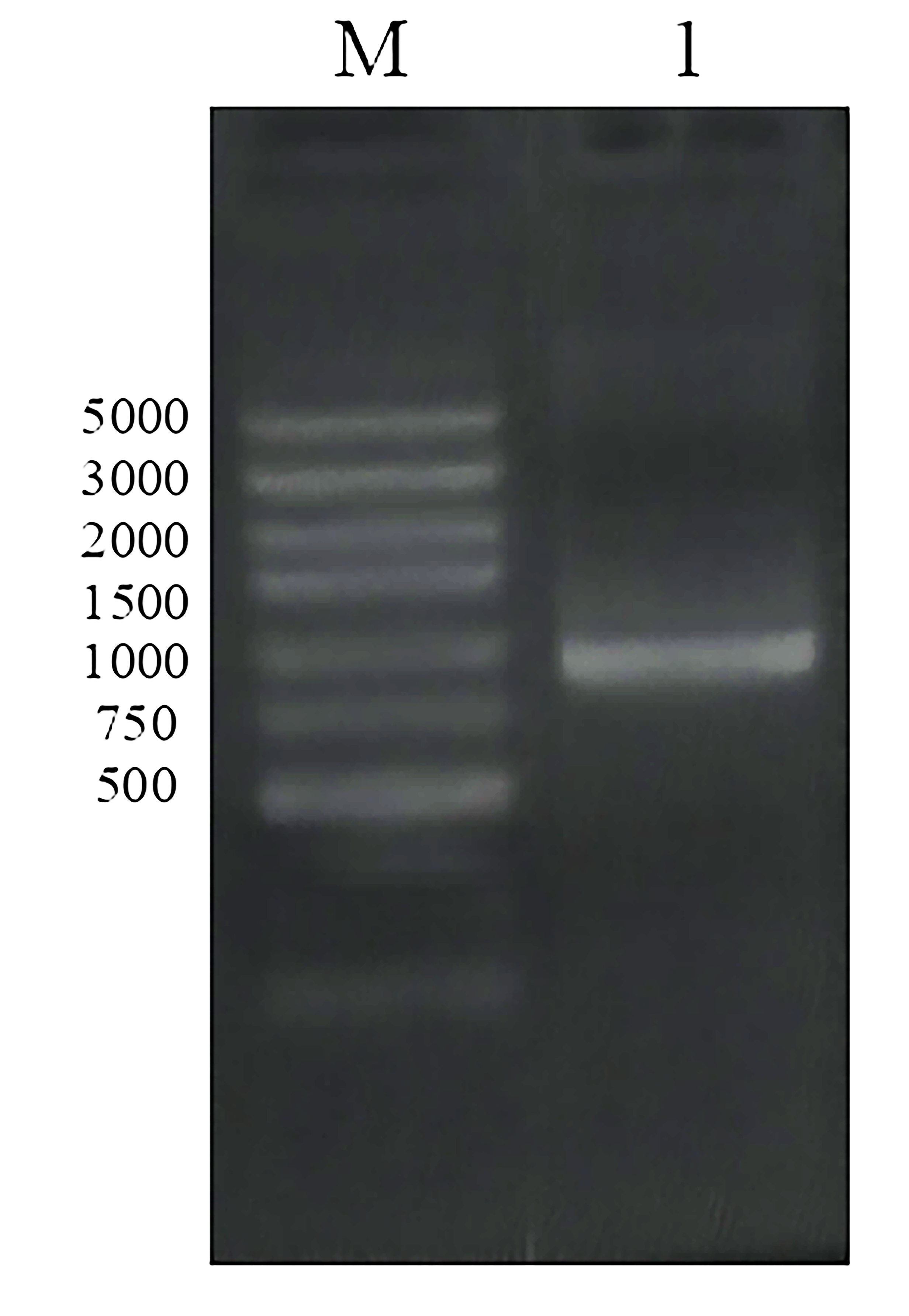

Supplement: Supplementary file 1 [file Image_1.TIF]

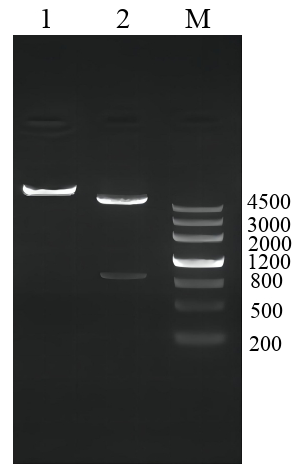

Supplement: Supplementary file 2 [file Image_2.TIF]
